# 1-Benzoyl­naphthalene-2,7-diyl dibenzoate

**DOI:** 10.1107/S1600536812052026

**Published:** 2013-01-09

**Authors:** Rei Sakamoto, Kosuke Sasagawa, Daichi Hijikata, Akiko Okamoto, Noriyuki Yonezawa

**Affiliations:** a Department of Organic and Polymer Materials Chemistry, Tokyo University of Agriculture & Technology 2-24-16 Naka-machi, Koganei, Tokyo 184-8588, Japan

## Abstract

In the title compound, C_31_H_20_O_5_, the phenyl rings of the benzo­yloxy and benzoyl groups are twisted away from the naphthalene ring system by 64.27 (6), 73.62 (5) and 80.41 (6)°. In the crystal, C—H⋯O hydrogen bonds and C—H⋯π inter­actions link the mol­ecules, forming tubular chains parallel to the *b* axis. The chains are further connected into a three-dimensional network by C—H⋯π inter­actions and π–π stacking contacts [centroid–centroid distances = 3.622 (10)–3.866 (12) Å].

## Related literature

For electrophilic aromatic aroylation of the naphthalene core, see: Okamoto & Yonezawa (2009 ▶); Okamoto *et al.* (2011 ▶). For structures of closely related compounds, see: Kato *et al.* (2010 ▶); Muto *et al.* (2011 ▶); Nakaema *et al.* (2008 ▶); Sakamoto *et al.* (2012 ▶); Watanabe *et al.* (2010 ▶).
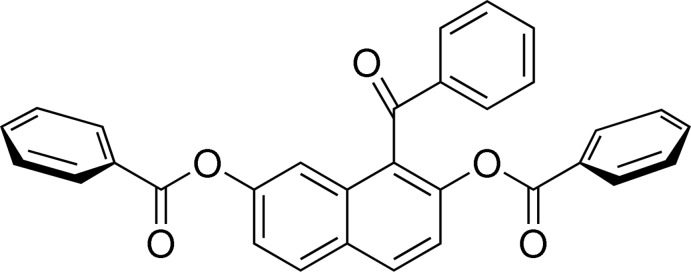


## Experimental

### 

#### Crystal data

C_31_H_20_O_5_
*M* *_r_* = 472.47
Monoclinic, 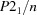

*a* = 16.1318 (3) Å
*b* = 7.18561 (13) Å
*c* = 20.7333 (4) Å
β = 99.180 (1)°
*V* = 2372.56 (7) Å^3^
*Z* = 4
Cu *K*α radiation
μ = 0.73 mm^−1^
*T* = 193 K
0.40 × 0.40 × 0.30 mm

#### Data collection

Rigaku R-AXIS RAPID diffractometer
Absorption correction: numerical (*NUMABS*; Higashi, 1999 ▶) *T*_min_ = 0.759, *T*_max_ = 0.811
40057 measured reflections
4282 independent reflections
3753 reflections with *I* > 2σ(*I*)
*R*_int_ = 0.027

#### Refinement

*R*[*F* ^2^ > 2σ(*F* ^2^)] = 0.037
*wR*(*F* ^2^) = 0.103
*S* = 1.07
4282 reflections
326 parameters
H-atom parameters constrained
Δρ_max_ = 0.18 e Å^−3^
Δρ_min_ = −0.16 e Å^−3^

### 

Data collection: *PROCESS-AUTO* (Rigaku, 1998 ▶); cell refinement: *PROCESS-AUTO*; data reduction: *CrystalStructure* (Rigaku, 2010 ▶); program(s) used to solve structure: *SIR2004* (Burla *et al.*, 2005 ▶); program(s) used to refine structure: *SHELXL97* (Sheldrick, 2008 ▶); molecular graphics: *ORTEPIII* (Burnett & Johnson, 1996 ▶); software used to prepare material for publication: *SHELXL97*.

## Supplementary Material

Crystal structure: contains datablock(s) I, global. DOI: 10.1107/S1600536812052026/rz5035sup1.cif

Structure factors: contains datablock(s) I. DOI: 10.1107/S1600536812052026/rz5035Isup2.hkl

Supplementary material file. DOI: 10.1107/S1600536812052026/rz5035Isup3.cml

Additional supplementary materials:  crystallographic information; 3D view; checkCIF report

## Figures and Tables

**Table 1:** Hydrogen-bond geometry (Å, °) *Cg*1 and *Cg*2 are the centroids of the C26–C31 and C5–C10 rings, respectively.

*D*—H⋯*A*	*D*—H	H⋯*A*	*D*⋯*A*	*D*—H⋯*A*
C3—H3⋯O1^i^	0.95	2.30	3.2183 (16)	164
C14—H14⋯O5^ii^	0.95	2.60	3.520 (2)	164
C29—H29⋯*Cg*1^iii^	0.95	2.72	3.6396 (18)	162
C15—H15⋯*Cg*2^ii^	0.95	2.78	3.6397 (19)	150

Symmetry codes: (i) 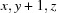; (ii) 
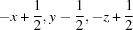
; (iii) 
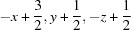
.

**Fig. 1.**
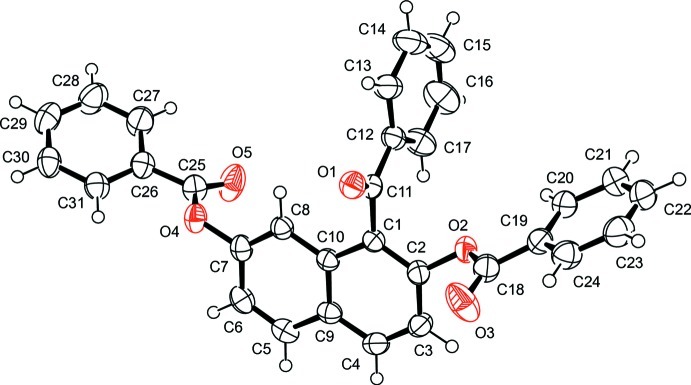
The molecular structure of the title compound with displacement ellipsoids drawn at the 50% probability level.

**Fig. 2.**
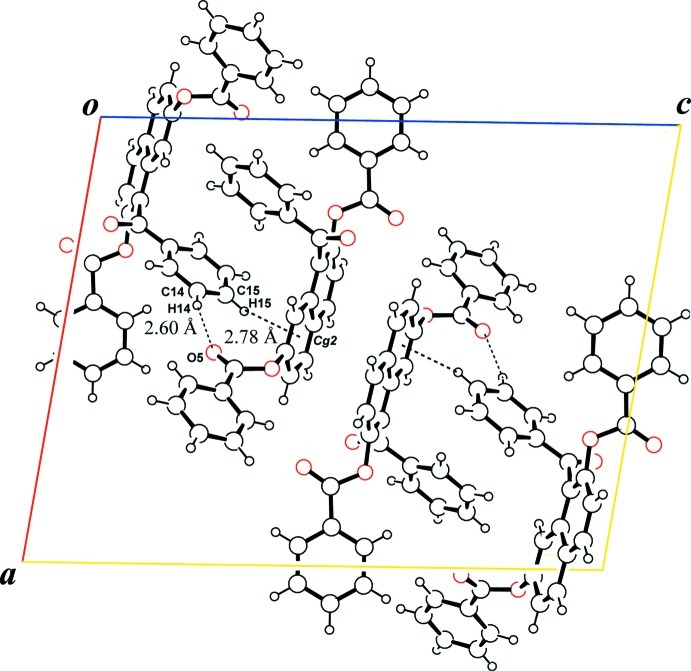
Partial crystal packing of the title compound showing C—H···O and C—H···π interactions as dashed lines.

**Fig. 3.**
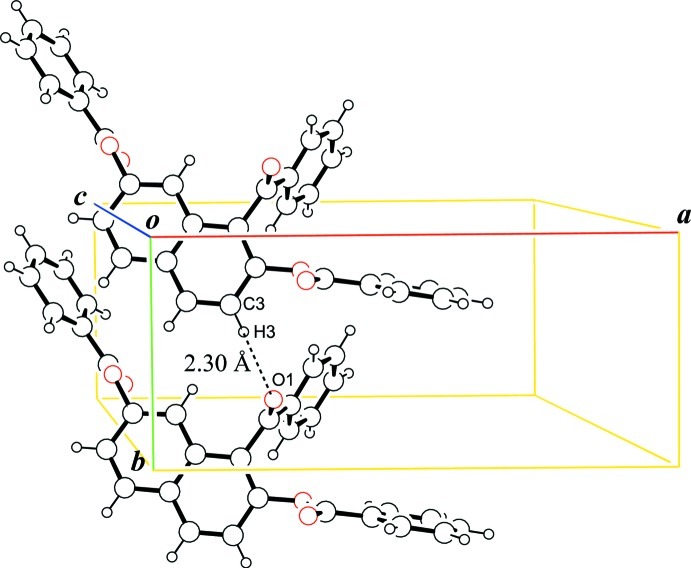
C–H···O interaction (dashed line) between naphthalene ring and ketonic carbonyl group.

## supplementary crystallographic information

### Comment

In the course of our study on electrophilic aromatic aroylation of the naphthalene core, 1,8-diaroylnaphthalene compounds have proved to be formed regioselectively by the aid of a suitable acidic mediator (Okamoto & Yonezawa, 2009, Okamoto *et al.*, 2011). Recently, we have reported the X-ray crystal structures of 1,8-diaroylnaphthalenes, *e.g.*, 1,8-dibenzoyl-2,7-dimethoxynaphthalene (Nakaema *et al.*, 2008). The two aroyl groups at 1,8-positions of the naphthalene ring in these compounds are perpendicularly attached to the naphthalene ring and oriented in opposite directions. Furthermore, we have also clarified the crystal structures of 1-monoaroylated naphthalene compounds such as (2,7-dimethoxynaphthalene-1-yl)-(phenyl)methanone (Kato *et al.*, 2010) and 2,7-dimethoxy-1-(4-nitrobenzoyl)naphthalene (Watanabe *et al.*, 2010). These compounds also exhibit essentially the same non-coplanar structure as the 1,8-diaroylated naphthalenes. Besides, the crystal structures of the aroylnaphthalene derivatives bearing benzoic ester moiety at the 2- or 2,7-positions on the naphthalene ring, 7-methoxy-1-(4-nitrobenzoyl)naphthalene-2-yl 4-nitrobenzoate (Muto *et al.*, 2011) and 1,8-dibenzoylnaphthalene-2,7-diyl dibenzoate (Sakamoto *et al.*, 2012) have been revealed.

The molecular structure of the title compound is displayed in Fig 1. The benzene ring of the benzoyl group is almost orthogonal to the naphthalene ring system forming a dihedral angle of 80.41 (6)° [C10—C1—C11—O1 torsion angle = 83.35 (15)°]. The two carbonyl moieties of the benzoyloxy groups at the 2,7-positions of the naphthalene ring system are in opposite directions relative to one another, as observed in the homologous compound 1,8-dibenzoylnaphthalene-2,7-diyl dibenzoate (Sakamoto *et al.*, 2012). The phenyl ring of the benzoyloxy group at the 7-position is inclined to form a narrower dihedral angle with the naphthalene ring system [64.27 (6)° and O5—C25—C26—C27 torsion angle = -21.8 (2)°] than the phenyl ring of the benzoyloxy group at the 2-position adjacent to the benzoyl group [73.62 (5)° and O3—C18—C19—C24 torsion angle = -2.1 (2)°]. In the crystal, C–H···O hydrogen bonds between an hydrogen atom of the phenyl ring of the benzoyl group and a carbonyl oxygen of the benzoyloxy group and between an hydrogen atom of the naphthalene ring system and a carbonyl oxygen of the benzoyl group, and weak C–H···π interactions (Table 1) link the molecules into tubular chains running parallel to the *b* axis (Fig. 2 and 3). Furthermore, the chains are connected into a three-dimensional network by weak C–H···π interactions [C15–H15···*Cg*2 = 2.78 Å; *Cg*2 is the centroids of the C5–C10 ring] and π–π contacts [*Cg*2^iii^···*Cg*3, 3.622 (10) Å; *Cg*4···*Cg*4^iv^ = 3.821 (12) Å; *Cg*4···*Cg*4^v^ = 3.866 (12) Å; *Cg*3 and *Cg*4 are the centroids of the C1/C4-C9-C10 and C19/C24 rings, respectively; symmetry codes: (iii) 1-x, 1-y, 1-z; (iv) -x, 1-y, 1-z; (v) -x, 2-y, 1-z).

### Experimental

The title compound was prepared *via* condensation reaction of 1-benzoyl-2,7-dihydroxynaphthalene (0.2 mmol, 52.86 mg) obtained by ethyl ether cleavage reaction of 1-benzoyl-2,7-diethoxynaphthalene, benzoyl chloride (0.4 mmol, 0.046 ml), and triethylamine (0.4 mmol, 0.056 ml) in dichloromethane (2.5 ml). After the reaction mixture was stirred at rt for 2 h, it was poured into water (30 ml) and the mixture was extracted with CHCl_3_ (10 ml×3). The combined extracts were washed with brine. The organic layers thus obtained were dried over anhydrous MgSO_4_. The solvent was removed under reduced pressure to give cake. The crude product was purified by recrystallization from ethyl acetate–hexane (3:1 *v*/*v*) and colorless single crystals suitable for X-ray diffraction were obtained (isolated yield 36%).

Spectroscopic data: ^1^H NMR δ (400 MHz, CDCl_3_): 7.24–7.38 (4*H*, m), 7.45–7.54 (6*H*, m), 7.59 (1*H*, d, *J* = 2.4 Hz), 7.63 (1*H*, t, *J* = 14.8 Hz), 7.74 (2*H*, d, *J* = 7.6 Hz), 7.85 (2*H*, d, *J* = 7.2 Hz), 8.02 (1*H*, d, *J* = 9.2 Hz), 8.08 (1*H*, d, *J* = 9.2 Hz), 8.18 (2*H*, d, *J* = 7.2 Hz) p.p.m.. ^13^C NMR δ (75 MHz, CDCl_3_): 116.33, 121.37, 121.93, 127.64, 128.26, 128.38, 128.51, 128.61, 129.10, 129.52, 129.59, 129.76, 129.87, 130.13, 130.86, 132.06, 133.59, 133.66, 133.76, 137.49, 146.53, 150.09, 164.18, 164.95, 195.32 p.p.m.. IR (KBr): 1739 (OC=O), 1658 (C=O), 1596,1582, 1510 (Ar) cm^-1^. m.p. = 422.0–422.8 K. HRMS (m/*z*): [*M*+H]^+^ calcd. for C_31_H_20_O_5_, 473.1390, found, 473.1384.

### Refinement

All H atoms were found in a difference map and were subsequently refined as riding atoms, with C–H = 0.95 Å, and with *U*_iso_(H) = 1.2 *U*_eq_(C).

### Crystal data

**Table:**

C_31_H_20_O_5_	*F*(000) = 984
*M**_r_* = 472.47	*D*_x_ = 1.323 Mg m^−^^3^
Monoclinic, *P*2_1_/*n*	Cu *K*α radiation, λ = 1.54187 Å
Hall symbol: -P 2yn	Cell parameters from 33959 reflections
*a* = 16.1318 (3) Å	θ = 3.2–68.2°
*b* = 7.18561 (13) Å	µ = 0.73 mm^−^^1^
*c* = 20.7333 (4) Å	*T* = 193 K
β = 99.180 (1)°	Block, colorless
*V* = 2372.56 (7) Å^3^	0.40 × 0.40 × 0.30 mm
*Z* = 4	

### Data collection

**Table:**

Rigaku R-AXIS RAPID diffractometer	4282 independent reflections
Radiation source: fine-focus sealed tube	3753 reflections with *I* > 2σ(*I*)
Graphite monochromator	*R*_int_ = 0.027
Detector resolution: 10.000 pixels mm^-1^	θ_max_ = 68.2°, θ_min_ = 3.2°
ω scans	*h* = −19→19
Absorption correction: numerical (*NUMABS*; Higashi, 1999)	*k* = −8→8
*T*_min_ = 0.759, *T*_max_ = 0.811	*l* = −24→24
40057 measured reflections	

### Refinement

**Table:**

Refinement on *F*^2^	Secondary atom site location: difference Fourier map
Least-squares matrix: full	Hydrogen site location: inferred from neighbouring sites
*R*[*F*^2^ > 2σ(*F*^2^)] = 0.037	H-atom parameters constrained
*wR*(*F*^2^) = 0.103	*w* = 1/[σ^2^(*F*_o_^2^) + (0.0548*P*)^2^ + 0.4366*P*] where *P* = (*F*_o_^2^ + 2*F*_c_^2^)/3
*S* = 1.07	(Δ/σ)_max_ < 0.001
4282 reflections	Δρ_max_ = 0.18 e Å^−^^3^
326 parameters	Δρ_min_ = −0.16 e Å^−^^3^
0 restraints	Extinction correction: *SHELXL97* (Sheldrick, 2008), Fc^*^=kFc[1+0.001xFc^2^λ^3^/sin(2θ)]^-1/4^
Primary atom site location: structure-invariant direct methods	Extinction coefficient: 0.0025 (2)

### Special details

**Table:**

Geometry. All e.s.d.'s (except the e.s.d. in the dihedral angle between two l.s. planes) are estimated using the full covariance matrix. The cell e.s.d.'s are taken into account individually in the estimation of e.s.d.'s in distances, angles and torsion angles; correlations between e.s.d.'s in cell parameters are only used when they are defined by crystal symmetry. An approximate (isotropic) treatment of cell e.s.d.'s is used for estimating e.s.d.'s involving l.s. planes.
Refinement. Refinement of *F*^2^ against ALL reflections. The weighted *R*-factor *wR* and goodness of fit *S* are based on *F*^2^, conventional *R*-factors *R* are based on *F*, with *F* set to zero for negative *F*^2^. The threshold expression of *F*^2^ > σ(*F*^2^) is used only for calculating *R*-factors(gt) *etc*. and is not relevant to the choice of reflections for refinement. *R*-factors based on *F*^2^ are statistically about twice as large as those based on *F*, and *R*- factors based on ALL data will be even larger.

### Fractional atomic coordinates and isotropic or equivalent isotropic displacement parameters (Å^2^)

**Table:**

	*x*	*y*	*z*	*U*_iso_*/*U*_eq_	
O1	0.26313 (6)	0.21093 (12)	0.46194 (4)	0.0438 (2)	
O2	0.20361 (5)	0.66287 (13)	0.43467 (4)	0.0425 (2)	
O3	0.21367 (7)	0.6819 (2)	0.54365 (5)	0.0750 (4)	
O4	0.56034 (6)	0.12325 (13)	0.37018 (4)	0.0434 (2)	
O5	0.52644 (9)	0.19992 (17)	0.26435 (5)	0.0712 (4)	
C1	0.32080 (7)	0.47803 (17)	0.42157 (6)	0.0336 (3)	
C2	0.29093 (8)	0.64552 (18)	0.43808 (6)	0.0378 (3)	
C3	0.34234 (9)	0.80265 (18)	0.45251 (6)	0.0424 (3)	
H3	0.3195	0.9172	0.4644	0.051*	
C4	0.42574 (9)	0.78701 (17)	0.44913 (6)	0.0405 (3)	
H4	0.4613	0.8917	0.4596	0.049*	
C5	0.54650 (8)	0.60238 (19)	0.42507 (6)	0.0409 (3)	
H5	0.5828	0.7056	0.4362	0.049*	
C6	0.57842 (8)	0.4414 (2)	0.40428 (6)	0.0426 (3)	
H6	0.6362	0.4326	0.4005	0.051*	
C7	0.52458 (8)	0.28923 (18)	0.38861 (6)	0.0376 (3)	
C8	0.44177 (8)	0.29459 (17)	0.39456 (6)	0.0350 (3)	
H8	0.4074	0.1878	0.3845	0.042*	
C9	0.46055 (8)	0.61883 (17)	0.43044 (6)	0.0354 (3)	
C10	0.40728 (7)	0.46095 (16)	0.41582 (5)	0.0327 (3)	
C11	0.26389 (7)	0.31006 (17)	0.41439 (6)	0.0341 (3)	
C12	0.21077 (8)	0.27099 (18)	0.35081 (6)	0.0386 (3)	
C13	0.16406 (9)	0.1071 (2)	0.34343 (7)	0.0495 (3)	
H13	0.1692	0.0191	0.3780	0.059*	
C14	0.11042 (10)	0.0725 (3)	0.28590 (8)	0.0637 (5)	
H14	0.0785	−0.0391	0.2809	0.076*	
C15	0.10333 (11)	0.1994 (3)	0.23606 (8)	0.0713 (5)	
H15	0.0652	0.1768	0.1970	0.086*	
C16	0.15089 (12)	0.3593 (3)	0.24206 (8)	0.0729 (5)	
H16	0.1466	0.4446	0.2067	0.088*	
C17	0.20496 (10)	0.3964 (2)	0.29958 (7)	0.0544 (4)	
H17	0.2377	0.5068	0.3038	0.065*	
C18	0.17106 (9)	0.68610 (19)	0.49101 (7)	0.0450 (3)	
C19	0.07915 (8)	0.71619 (18)	0.47694 (7)	0.0413 (3)	
C20	0.03506 (8)	0.72662 (19)	0.41374 (7)	0.0433 (3)	
H20	0.0637	0.7108	0.3775	0.052*	
C21	−0.05051 (9)	0.7600 (2)	0.40370 (8)	0.0506 (4)	
H21	−0.0807	0.7679	0.3605	0.061*	
C22	−0.09207 (9)	0.7819 (2)	0.45659 (8)	0.0556 (4)	
H22	−0.1507	0.8059	0.4497	0.067*	
C23	−0.04879 (10)	0.7690 (2)	0.51924 (8)	0.0560 (4)	
H23	−0.0778	0.7826	0.5554	0.067*	
C24	0.03664 (10)	0.7363 (2)	0.52965 (7)	0.0505 (4)	
H24	0.0664	0.7276	0.5730	0.061*	
C25	0.56198 (8)	0.0984 (2)	0.30540 (6)	0.0439 (3)	
C26	0.61134 (8)	−0.06865 (19)	0.29340 (6)	0.0415 (3)	
C27	0.59531 (11)	−0.1511 (2)	0.23219 (8)	0.0579 (4)	
H27	0.5543	−0.0993	0.1991	0.069*	
C28	0.63909 (12)	−0.3084 (2)	0.21950 (9)	0.0670 (5)	
H28	0.6277	−0.3655	0.1777	0.080*	
C29	0.69917 (10)	−0.3831 (2)	0.26699 (8)	0.0577 (4)	
H29	0.7287	−0.4921	0.2581	0.069*	
C30	0.71647 (9)	−0.2999 (2)	0.32736 (8)	0.0500 (4)	
H30	0.7587	−0.3505	0.3598	0.060*	
C31	0.67265 (8)	−0.1426 (2)	0.34107 (7)	0.0440 (3)	
H31	0.6845	−0.0857	0.3829	0.053*	

### Atomic displacement parameters (Å^2^)

**Table:**

	*U*^11^	*U*^22^	*U*^33^	*U*^12^	*U*^13^	*U*^23^
O1	0.0459 (5)	0.0411 (5)	0.0436 (5)	0.0008 (4)	0.0050 (4)	0.0052 (4)
O2	0.0361 (5)	0.0463 (5)	0.0443 (5)	0.0103 (4)	0.0037 (4)	−0.0065 (4)
O3	0.0498 (6)	0.1293 (12)	0.0434 (6)	0.0096 (7)	0.0000 (5)	0.0035 (6)
O4	0.0428 (5)	0.0494 (5)	0.0397 (5)	0.0133 (4)	0.0120 (4)	0.0041 (4)
O5	0.1021 (9)	0.0679 (7)	0.0420 (6)	0.0397 (7)	0.0066 (6)	0.0050 (5)
C1	0.0337 (6)	0.0344 (6)	0.0314 (6)	0.0018 (5)	0.0014 (5)	−0.0007 (5)
C2	0.0354 (6)	0.0390 (7)	0.0376 (6)	0.0052 (5)	0.0020 (5)	−0.0014 (5)
C3	0.0509 (8)	0.0329 (6)	0.0413 (7)	0.0048 (5)	0.0015 (6)	−0.0033 (5)
C4	0.0486 (8)	0.0346 (7)	0.0359 (6)	−0.0053 (5)	−0.0009 (5)	−0.0003 (5)
C5	0.0384 (7)	0.0480 (7)	0.0351 (6)	−0.0088 (6)	0.0022 (5)	0.0047 (5)
C6	0.0334 (6)	0.0551 (8)	0.0398 (7)	−0.0001 (6)	0.0074 (5)	0.0081 (6)
C7	0.0379 (7)	0.0433 (7)	0.0320 (6)	0.0068 (5)	0.0063 (5)	0.0042 (5)
C8	0.0348 (6)	0.0366 (6)	0.0330 (6)	0.0007 (5)	0.0037 (5)	0.0006 (5)
C9	0.0387 (7)	0.0372 (6)	0.0291 (6)	−0.0037 (5)	0.0013 (5)	0.0027 (5)
C10	0.0338 (6)	0.0356 (6)	0.0276 (6)	0.0004 (5)	0.0016 (5)	0.0016 (5)
C11	0.0299 (6)	0.0349 (6)	0.0379 (6)	0.0056 (5)	0.0067 (5)	−0.0023 (5)
C12	0.0328 (6)	0.0457 (7)	0.0380 (7)	−0.0004 (5)	0.0074 (5)	−0.0069 (5)
C13	0.0448 (8)	0.0523 (8)	0.0518 (8)	−0.0068 (6)	0.0090 (6)	−0.0134 (7)
C14	0.0525 (9)	0.0782 (11)	0.0598 (10)	−0.0155 (8)	0.0068 (7)	−0.0281 (9)
C15	0.0553 (10)	0.1101 (15)	0.0453 (9)	−0.0065 (10)	−0.0017 (7)	−0.0271 (10)
C16	0.0739 (12)	0.1018 (15)	0.0393 (8)	−0.0034 (11)	−0.0025 (8)	0.0056 (9)
C17	0.0519 (8)	0.0668 (10)	0.0429 (8)	−0.0090 (7)	0.0031 (6)	0.0021 (7)
C18	0.0459 (8)	0.0463 (7)	0.0427 (7)	0.0049 (6)	0.0062 (6)	−0.0003 (6)
C19	0.0418 (7)	0.0378 (7)	0.0448 (7)	0.0010 (5)	0.0080 (6)	−0.0022 (5)
C20	0.0412 (7)	0.0434 (7)	0.0459 (7)	0.0023 (6)	0.0086 (6)	−0.0019 (6)
C21	0.0415 (7)	0.0532 (8)	0.0559 (9)	0.0024 (6)	0.0045 (6)	−0.0015 (7)
C22	0.0404 (8)	0.0544 (9)	0.0736 (10)	0.0009 (6)	0.0138 (7)	−0.0081 (8)
C23	0.0528 (9)	0.0593 (9)	0.0610 (9)	−0.0033 (7)	0.0246 (7)	−0.0117 (7)
C24	0.0519 (8)	0.0538 (8)	0.0470 (8)	−0.0025 (7)	0.0118 (7)	−0.0054 (6)
C25	0.0435 (7)	0.0481 (7)	0.0406 (7)	0.0051 (6)	0.0085 (6)	0.0029 (6)
C26	0.0393 (7)	0.0436 (7)	0.0440 (7)	0.0022 (6)	0.0141 (6)	0.0034 (6)
C27	0.0674 (10)	0.0573 (9)	0.0478 (8)	0.0123 (8)	0.0061 (7)	−0.0009 (7)
C28	0.0854 (13)	0.0589 (10)	0.0574 (10)	0.0143 (9)	0.0130 (9)	−0.0116 (8)
C29	0.0600 (9)	0.0462 (8)	0.0724 (11)	0.0096 (7)	0.0273 (8)	−0.0001 (7)
C30	0.0385 (7)	0.0516 (8)	0.0625 (9)	0.0070 (6)	0.0156 (7)	0.0083 (7)
C31	0.0370 (7)	0.0489 (8)	0.0480 (8)	0.0009 (6)	0.0130 (6)	0.0029 (6)

### Geometric parameters (Å, º)

**Table:**

O1—C11	1.2180 (15)	C14—H14	0.9500
O2—C18	1.3653 (17)	C15—C16	1.377 (3)
O2—C2	1.4047 (15)	C15—H15	0.9500
O3—C18	1.1938 (17)	C16—C17	1.386 (2)
O4—C25	1.3594 (16)	C16—H16	0.9500
O4—C7	1.4041 (15)	C17—H17	0.9500
O5—C25	1.1957 (16)	C18—C19	1.4805 (19)
C1—C2	1.3602 (17)	C19—C24	1.387 (2)
C1—C10	1.4243 (17)	C19—C20	1.3897 (19)
C1—C11	1.5093 (17)	C20—C21	1.3837 (19)
C2—C3	1.4046 (18)	C20—H20	0.9500
C3—C4	1.363 (2)	C21—C22	1.382 (2)
C3—H3	0.9500	C21—H21	0.9500
C4—C9	1.4126 (18)	C22—C23	1.376 (2)
C4—H4	0.9500	C22—H22	0.9500
C5—C6	1.363 (2)	C23—C24	1.381 (2)
C5—C9	1.4136 (18)	C23—H23	0.9500
C5—H5	0.9500	C24—H24	0.9500
C6—C7	1.4021 (19)	C25—C26	1.4832 (19)
C6—H6	0.9500	C26—C27	1.387 (2)
C7—C8	1.3616 (18)	C26—C31	1.3876 (18)
C8—C10	1.4182 (17)	C27—C28	1.381 (2)
C8—H8	0.9500	C27—H27	0.9500
C9—C10	1.4262 (17)	C28—C29	1.375 (2)
C11—C12	1.4800 (17)	C28—H28	0.9500
C12—C17	1.384 (2)	C29—C30	1.375 (2)
C12—C13	1.3934 (19)	C29—H29	0.9500
C13—C14	1.380 (2)	C30—C31	1.386 (2)
C13—H13	0.9500	C30—H30	0.9500
C14—C15	1.369 (3)	C31—H31	0.9500
			
C18—O2—C2	119.17 (10)	C15—C16—H16	119.9
C25—O4—C7	117.01 (10)	C17—C16—H16	119.9
C2—C1—C10	119.22 (11)	C12—C17—C16	119.43 (15)
C2—C1—C11	119.88 (11)	C12—C17—H17	120.3
C10—C1—C11	120.75 (10)	C16—C17—H17	120.3
C1—C2—C3	122.96 (12)	O3—C18—O2	122.35 (13)
C1—C2—O2	117.29 (11)	O3—C18—C19	126.61 (14)
C3—C2—O2	119.51 (11)	O2—C18—C19	111.04 (11)
C4—C3—C2	118.48 (12)	C24—C19—C20	119.62 (13)
C4—C3—H3	120.8	C24—C19—C18	117.73 (13)
C2—C3—H3	120.8	C20—C19—C18	122.65 (13)
C3—C4—C9	121.57 (12)	C21—C20—C19	119.96 (13)
C3—C4—H4	119.2	C21—C20—H20	120.0
C9—C4—H4	119.2	C19—C20—H20	120.0
C6—C5—C9	121.36 (12)	C22—C21—C20	119.92 (14)
C6—C5—H5	119.3	C22—C21—H21	120.0
C9—C5—H5	119.3	C20—C21—H21	120.0
C5—C6—C7	118.93 (12)	C23—C22—C21	120.25 (14)
C5—C6—H6	120.5	C23—C22—H22	119.9
C7—C6—H6	120.5	C21—C22—H22	119.9
C8—C7—C6	122.50 (12)	C22—C23—C24	120.17 (15)
C8—C7—O4	120.02 (11)	C22—C23—H23	119.9
C6—C7—O4	117.35 (11)	C24—C23—H23	119.9
C7—C8—C10	119.43 (11)	C23—C24—C19	120.08 (14)
C7—C8—H8	120.3	C23—C24—H24	120.0
C10—C8—H8	120.3	C19—C24—H24	120.0
C4—C9—C5	122.07 (11)	O5—C25—O4	122.70 (13)
C4—C9—C10	119.10 (11)	O5—C25—C26	125.71 (13)
C5—C9—C10	118.82 (11)	O4—C25—C26	111.58 (11)
C8—C10—C1	122.46 (11)	C27—C26—C31	119.74 (13)
C8—C10—C9	118.92 (11)	C27—C26—C25	118.19 (12)
C1—C10—C9	118.60 (11)	C31—C26—C25	122.08 (12)
O1—C11—C12	122.06 (11)	C28—C27—C26	119.85 (15)
O1—C11—C1	118.18 (11)	C28—C27—H27	120.1
C12—C11—C1	119.75 (11)	C26—C27—H27	120.1
C17—C12—C13	119.77 (13)	C29—C28—C27	120.41 (16)
C17—C12—C11	121.24 (12)	C29—C28—H28	119.8
C13—C12—C11	118.96 (12)	C27—C28—H28	119.8
C14—C13—C12	120.03 (15)	C30—C29—C28	119.99 (14)
C14—C13—H13	120.0	C30—C29—H29	120.0
C12—C13—H13	120.0	C28—C29—H29	120.0
C15—C14—C13	119.88 (16)	C29—C30—C31	120.33 (14)
C15—C14—H14	120.1	C29—C30—H30	119.8
C13—C14—H14	120.1	C31—C30—H30	119.8
C14—C15—C16	120.63 (15)	C30—C31—C26	119.66 (14)
C14—C15—H15	119.7	C30—C31—H31	120.2
C16—C15—H15	119.7	C26—C31—H31	120.2
C15—C16—C17	120.21 (17)		
			
C10—C1—C2—C3	−2.69 (19)	C1—C11—C12—C13	174.58 (12)
C11—C1—C2—C3	172.90 (11)	C17—C12—C13—C14	−1.8 (2)
C10—C1—C2—O2	171.67 (10)	C11—C12—C13—C14	176.31 (13)
C11—C1—C2—O2	−12.74 (17)	C12—C13—C14—C15	0.1 (2)
C18—O2—C2—C1	112.74 (13)	C13—C14—C15—C16	1.7 (3)
C18—O2—C2—C3	−72.69 (16)	C14—C15—C16—C17	−1.8 (3)
C1—C2—C3—C4	0.7 (2)	C13—C12—C17—C16	1.7 (2)
O2—C2—C3—C4	−173.51 (11)	C11—C12—C17—C16	−176.40 (14)
C2—C3—C4—C9	1.19 (19)	C15—C16—C17—C12	0.1 (3)
C9—C5—C6—C7	−0.61 (19)	C2—O2—C18—O3	−4.3 (2)
C5—C6—C7—C8	−1.32 (19)	C2—O2—C18—C19	175.60 (11)
C5—C6—C7—O4	−177.23 (11)	O3—C18—C19—C24	−2.1 (2)
C25—O4—C7—C8	92.97 (14)	O2—C18—C19—C24	178.03 (12)
C25—O4—C7—C6	−91.01 (14)	O3—C18—C19—C20	177.25 (16)
C6—C7—C8—C10	1.59 (18)	O2—C18—C19—C20	−2.63 (18)
O4—C7—C8—C10	177.40 (10)	C24—C19—C20—C21	1.1 (2)
C3—C4—C9—C5	178.27 (12)	C18—C19—C20—C21	−178.25 (13)
C3—C4—C9—C10	−1.06 (18)	C19—C20—C21—C22	−0.4 (2)
C6—C5—C9—C4	−177.17 (12)	C20—C21—C22—C23	−0.5 (2)
C6—C5—C9—C10	2.16 (18)	C21—C22—C23—C24	0.8 (2)
C7—C8—C10—C1	178.38 (11)	C22—C23—C24—C19	0.0 (2)
C7—C8—C10—C9	0.03 (17)	C20—C19—C24—C23	−0.9 (2)
C2—C1—C10—C8	−175.64 (11)	C18—C19—C24—C23	178.49 (14)
C11—C1—C10—C8	8.81 (17)	C7—O4—C25—O5	−8.6 (2)
C2—C1—C10—C9	2.72 (17)	C7—O4—C25—C26	172.23 (11)
C11—C1—C10—C9	−172.83 (10)	O5—C25—C26—C27	−21.8 (2)
C4—C9—C10—C8	177.51 (10)	O4—C25—C26—C27	157.33 (13)
C5—C9—C10—C8	−1.84 (16)	O5—C25—C26—C31	157.80 (16)
C4—C9—C10—C1	−0.91 (16)	O4—C25—C26—C31	−23.11 (18)
C5—C9—C10—C1	179.74 (10)	C31—C26—C27—C28	1.4 (2)
C2—C1—C11—O1	−92.16 (14)	C25—C26—C27—C28	−179.01 (15)
C10—C1—C11—O1	83.36 (14)	C26—C27—C28—C29	−0.6 (3)
C2—C1—C11—C12	87.46 (14)	C27—C28—C29—C30	−0.6 (3)
C10—C1—C11—C12	−97.02 (13)	C28—C29—C30—C31	1.1 (2)
O1—C11—C12—C17	172.31 (13)	C29—C30—C31—C26	−0.3 (2)
C1—C11—C12—C17	−7.30 (18)	C27—C26—C31—C30	−0.9 (2)
O1—C11—C12—C13	−5.81 (18)	C25—C26—C31—C30	179.50 (12)

### Hydrogen-bond geometry (Å, º)

Cg1 and Cg2 are the centroids of the C26–C31 and C5–C10 rings, respectively.

**Table:**

*D*—H···*A*	*D*—H	H···*A*	*D*···*A*	*D*—H···*A*
C3—H3···O1^i^	0.95	2.30	3.2183 (16)	164
C14—H14···O5^ii^	0.95	2.60	3.520 (2)	164
C29—H29···*Cg*1^iii^	0.95	2.72	3.6396 (18)	162
C15—H15···*Cg*2^ii^	0.95	2.78	3.6397 (19)	150

Symmetry codes: (i) *x*, *y*+1, *z*; (ii) −*x*+1/2, *y*−1/2, −*z*+1/2; (iii) −*x*+3/2, *y*+1/2, −*z*+1/2.
